# Marketing health education: advertising margarine and visualising health in Britain from 1964–c.2000

**DOI:** 10.1080/13619462.2017.1305898

**Published:** 2017-04-11

**Authors:** Jane Hand

**Affiliations:** ^a^ History, University of Warwick, Coventry, UK

**Keywords:** Margarine, advertising, visual culture, gender, heart disease

## Abstract

During the post-war period, margarine was re-conceptualised as a value-added product with distinct health benefits. This article contextualises the advertising of margarine as a healthy food, focusing on Unilever’s Flora brand as an important case study in legitimising the emergent role of disease prevention as a marketing tool. It uses the methodology of visual culture to examine how advertising employed chronic disease prevention as a selling tool. This article assesses how the post-war environment gave rise to new ways of visually advertising food, and how these promoted innovative visualisations of food, the body and their interactions with health.

## Introduction

There has been a general assumption by some historians that margarine as a low-cost, poor value alternative to butter never gained significant consumer ground until it could be sold in terms of health from the 1980s onwards.^1^ However, this article argues that throughout the post-war period, margarine was undergoing a slow but significant reorientation around the concept of health and healthiness. This transition from margarine as a low-cost but inferior butter substitute to a product with important distinctive benefits certainly gained credence from the increased scientific assumption that natural, animal fats might be playing a major role in the increase in coronary heart disease (CHD) in Britain during the post-war period.^2^ The margarine industry was increasingly committed to utilising health as a means of creating market segmentation separate from ongoing concerns about flavour and nutritive quality throughout most of the second half of the twentieth century.^3^ In particular, the Flora margarine brand manufactured by Unilever PLC was the first to be marketed and sold in terms of its potential health benefits.^4^ This identification of health by Unilever, especially from the 1960s, would determine the course of its margarine marketing for the remainder of the century as ever-increasing efforts to scientifically link margarine consumption with reduced risk of CHD took hold.

Much academic research investigating the positioning of margarine in Britain since the late nineteenth century has emphasised the close associations between margarine consumption and class, alongside conceptions of poverty and inferiority.^5^ Alysa Levene has argued that even after the nutritive quality and flavour of margarine was greatly improved by the middle of the twentieth century, the understanding of it as an inferior product persisted.^6^ She reasoned that because margarine was intimately bound up with austerity and rationing, it remained difficult for manufacturers to overcome its traditional associations with poverty, the working-classes and low nutritional value (when compared with butter). Her assertion that the 1980s marked the ‘turning point’ for margarine in that it could now be advertised and sold in terms of disease prevention is however to underestimate the shift in advertising focus that margarine underwent in the previous three decades. Because Levene’s study focused largely on analysing the opinions of respondents to Mass-Observation surveys during the 1930s and 1950s, her work is less concerned with the role that the food industry and product advertising more generally played in reorienting margarine away from wartime austerity and towards notions of modernity and health. This article will redress the continued scholarly neglect of visual advertising and the role of the food industry in selling margarine as a product with inherent positive values before this apparent ‘turning point’ in the 1980s.

I employ the methodologies of visual culture, an interdisciplinary approach that critically interprets the meaning of images, in order to analyse the advertising output of Unilever PLC in visual terms. I focus mainly on their post-war margarine brand Flora because it re-constructed margarine as a product that both epitomised modernism and linked this modernity with the acquisition of bodily healthiness. I examine in particular a selection of newspaper and magazine advertisements, produced by the Unilever-owned advertising agency Lintas, to suggest that far from being marketed as a straightforward butter replacement, margarine was undergoing a rapid transformation during the 1960s and 1970s. This transition paved the way for the marketing of margarine that not only contained polyunsaturated fats but also plant sterols (cholesterol-lowering substances naturally occurring in small amounts in many grains, fruit and vegetables added to margarine during manufacturing) and brands such as Flora ProActiv by the end of the twentieth century.

This article links approaches in the social history of medicine with the field of visual studies to analyse the ways images circulated knowledge about diet, disease, gender and the body. I historicise these images as important vehicles of information provision, reclaiming their place in the historical understanding of risk and the body in post-war Britain. The increased epistemological status given to images in historical analysis has influenced histories of public health in recent years, emphasising the importance of the visual in establishing public awareness of disease.^7^ Such aspects of health campaigns pertaining to nutrition and health have ultimately become central to wider investigations into the cultural perceptions of disease, the body and gender relations, both past and present.

This research utilises a case-study approach for analysing the contribution of commercial enterprise to the visualisation of diet and disease risk. This is necessitated not only because of the patchy source base available to the historian in examining the place of individual campaigns within the broader advertising culture of the post-war period, but also because the focus on one individual food producer allows for the elevation of images and imagistic cultures that would remain under-explored in a study with a wide focus. I concentrate on Unilever as a distinct example because of its position as a leading food producer in post-war Britain. Unilever was the first margarine producer to identify health as an important value-added component allied to brand diversification in the post-war period. Importantly, it was also the first to develop, produce, launch and market a polyunsaturated margarine brand—Flora—that claimed particular health benefits for the consumer. Flora was one of the first ‘soft’ margarines to be launched in Britain and by 1973 such ‘soft’ margarines had captured 65 per cent of the volume and 75 per cent of the value of margarine sales in Britain. Competitors such as Kraft Soft and Kraft Carousel were launched in 1973 and in response Unilever increased its advertising share to 95 per cent, once more dominating the market.^8^ The National Food Survey indicated that while standard margarine brands remained unpopular, Flora performed well as a healthy margarine throughout the 1970s and 1980s.^9^

Approaching the history of healthy eating in post-war Britain from the viewpoint of visual advertising raises its own particular problems. There is a general question of seeing visual advertising as an imperfect source whereby issues of reception and change are difficult to measure and analyse. Even after the introduction of consumer surveys and other forms of examining ‘success’ in the post-war period, there remained a disconnect between advertisements and consumer responses. While some historians have championed an approach centred on viewing large numbers of advertisements to locate patterns that ‘reflect common ways of “crafting and of seeing imagery” in a given time period’, I would argue that this is reductive in that it views images merely in terms of patterns of representation—the search for a ‘visual hegemony’—rather than examining advertisements as idiosyncratic cultural products that often employ disparate and unique visual formulae to ‘sell’ ideas, desires and lifestyles.^10^ Rather than arguing for a ‘visual hegemony’, it is more useful to acknowledge that images emitted differing overt and covert messages, themselves culturally contingent and to emphasise their subject matter or thematic structure as linking devices.

My selection of visual advertisements for analysis reflects the ephemeral nature of these sources and the extant examples available through private and commercial archives. While I have been unable to access background information due to archival limitations on campaign planning in the case of Flora, I have been able to piece together a corpus of images that represent Unilever’s national Flora advertising campaigns from its first introduction in Britain in 1964 until the late 1980s. These advertisements characterise the visual shift from ‘traditional’ female-centred advertisements during the Flora test phase, through the decision to focus on the visual representation of the at-risk male, widening out to all males and finally re-incorporating the female purchaser and consumer. This has enabled me to undertake a close reading of these advertisements, and their visual components.

My visual methodology necessitates the use of particular examples as expressive objects that advance specific understandings of diet and disease and as such I do not employ methods of textual content analysis. Instead, I use visual theory to tie these images to Unilever’s changing concerns over market segmentation, consumer base and the role of print advertising within British advertising culture. This study does not make a claim for the place of Unilever’s margarine products within the broader context of butter marketing, in part because the health implications of the former were increasingly at odds with the latter. Instead, its specificity exposes how Unilever’s branded margarine product Flora utilised its differentness from butter, coded explicitly in health terms, to examine how one commercial entity appropriated the emerging tenets of risk, individualism and behavioural change in order to sell a food product. I argue that within the new post-war commercial world, Unilever was able to play a significant role in circulating information regarding food and health to the population.

In the post-war period, nutrition science began to concentrate on understanding the proliferation of chronic disease within the context of risk-factor epidemiology.^11^ A range of scientific studies, especially the Framingham Study and the Seven Countries Study suggested a strong correlation between diets high in saturated fat and increased risk of heart disease. In conjunction with this elevation of diet as a risk factor for CHD within the scientific community, the rise of expert committees as important components of governmental policy-making procedure allowed scientific ideas and the findings of randomised control trials to be distilled (albeit with variable degrees of efficacy) into governmental health policy.^12^ A new public health conversation around diet and saturated fat as risk factors for CHD emerged, which emphasised lifestyle and individual behaviour as key agents in disease risk. Thus, as scientific evidence made it increasingly possible that ‘natural’ dairy fats were having an adverse effect on heart health, the potential to align ‘synthetic’ margarine with a positive health message enabled Unilever to launch and market Flora as the margarine for a healthy heart from the 1960s. Through a close visual analysis of a number of advertisements, this article will argue for the role of margarine in promoting disease prevention as a marketing tool in the post-war period. I also suggest that understandings of margarine in relation to class and poverty were increasingly irrelevant to the advertising construction of margarine during this period. Instead, the image of post-war margarine was formed around very different schemata than its wartime predecessors. Modernity and the rise of chronic illness were, I would suggest, more commonly visualised factors that impacted on the development of margarine and its close associations with health in advertising terms rather than class, social status or any association with poverty.

I will examine the emergence of Flora margarine in relation to chronic disease prevention, arguing for the role of images in transmitting knowledge about the impact of diet on heath. I argue that by utilising a health education outlook within its visual advertisements, Unilever was committed to providing an important ancillary source of health education information to the public while exploiting contemporary understandings of gender and the body to accentuate this. Finally, I draw this narrative of margarine marketing in relation to health together and suggest that it was this long post-war history of aligning margarine with health that facilitated the emergence of Flora ProActiv. By approaching the story through the lens of published visual advertising, this article hopes to move beyond concerns with class and frugality alone in order to allow margarine to be historicised within the wider context of consumer culture around healthy eating and dieting.

## Unilever and a chronic disease prevention agenda

With the end of food rationing in 1954, Unilever re-launched many of their margarine brands that had been suspended during the war and introduced new brands with qualities, such as spreadability from the fridge that had eluded pre-War production technologies.^13^ While the edible fats trade experienced a short-lived consumer boom in the mid-1950s, sales quickly levelled off. As a result, Unilever began investing in diversification to enable sustained market segmentation. One area that it became committed to was aligning margarine with health.^14^ An implicit language of health had been applied to Unilever’s margarine brands from the mid-1950s until the mid-1960s, but it was not until the nationwide launch of Flora margarine in 1968 that Unilever explicitly linked health with a brand identity.^15^ As such Flora was the first margarine brand advertised as a product with specific health benefits drawn from their high-polyunsaturated fat content.^16^

As medical science slowly identified the potential role of saturated fats in the likelihood of developing CHD, Unilever seized on societal reactions by engaging at-risk individuals as key agents of behavioural change, empowering them through consumption and the commodification of disease prevention. From 1969, Flora margarine advertisements focused on the visual image of men—those typically at high risk from CHD—to market the margarine as a health product.^17^ This marked a distinct departure for Unilever in its margarine advertising, which had hitherto almost invariably represented women—the principal food shopper. Thus, by marketing the brand not only in relation to gender but also to health, they capitalised on a wider consumer interest in diet, slimming and fitness.^18^

With their Flora margarine advertisements, Unilever firmly aligned healthy eating habits with both masculinity and modernity. Feminist scholarly work has repeatedly emphasised the pressure on women to subscribe to a vast array of socially constructed bodily ‘norms’.^19^ Yet the cultural practices of dieting, fitness and image management worked together with a post-war image culture that encouraged firstly women, but later men, to see themselves and their personal appearance as inadequate and consequently in need of improvement.^20^ While the narrative of modernity during the twentieth century has received committed scholarly attention as it impacted upon women, it is only more recently that men have been re-integrated into understandings of gender relations in more nuanced ways.^21^ These studies have shown that changes (while less radical) were also occurring in how men were imagined, represented and discussed in terms of beauty, the body and the presentation of maleness (and masculinity) in everyday life. Certainly, the marketing of Flora emphasised that advertising health was closely related to a keep-fit culture primarily concerned with looking attractive. Rather than emphasising the body at risk from disease, the male body was portrayed as fit and healthy. The ideal physique (and thus spectacle) of the ‘perfect’ male body has a dual meaning in part defined by what it is not—the ‘perfect’ body also implies its opposite, the imperfect, the unhealthy or decayed body.^22^ Rather than emphasising the ‘ugly’, unattractive body to instigate behavioural (or in this case purchasing) change on the part of the consumer, the marketing of Flora utilised the attractive body (or at least attractive body parts) for the same end.

Flora as a case study, therefore, offers interesting points of continuity and difference in the ways health, disease, diet and the body were represented visually in the post-war period. It dovetails with contemporary governmental campaigns, yet for very different purposes. With body weights in Britain steadily rising from the 1960s, so too were associated diseases, particularly heart disease. Risk-factor epidemiology suggested a strong correlation between diets high in saturated fat and the increased incidence of CHD. Within this context, the government promoted risk-avoiding behaviour as a fundamental element of its public health agenda. In particular, the Health Education Council appropriated an important role in publicising healthy behaviours to the population. Their health education campaigns during this period were focused on encouraging healthy eating and more exercise and used gendered ideals of bodily beauty and attractiveness to publicise this.^23^ Yet, the use of commercial advertising to similarly promote health in terms of the body by Unilever represents an interesting crossover between government and industry in post-war Britain.

From the outset, Flora was coded as a health-promoting product. An important component of Unilever’s strategy for marketing Flora was ‘to enlighten … the general public as to the facts regarding dietary fats and heart disease’.^24^ By their own admission, Unilever established Flora as not just a brand but as a type of ‘health expert’ that ‘made Flora advice you’ll take on board’.^25^ Certainly, what set Flora apart from its competitors in this instance was Unilever’s apparent commitment to fulfilling a health education function. This dedication was realised in pragmatic terms with the establishment in 1971 of the Flora Information Service, which provided information about heart disease and dietary fats to the public. In part a public relations project for the Flora brand, the Information Service researched, produced and distributed advice literature mainly for health and education professionals.^26^ In 1977, the Service’s name was changed to the Flora Project for Heart Disease Prevention with the Flora Information Service continuing to act as its literature arm. This reflected the widening scope of the Project with activities focused on lectures, conferences, exhibitions for health professionals and events to target school children. The establishment of this Service reflected a wider evolution of infrastructure within multinational companies associated with corporate social responsibility. While social responsibility was beginning to feature on the agenda of large corporations during the inter-war period, from the 1960s it was routinely incorporated into brand strategies. Such initiatives were important in the creation of a consumer culture around health, disease and consumer action.^27^

**Figure 1.**
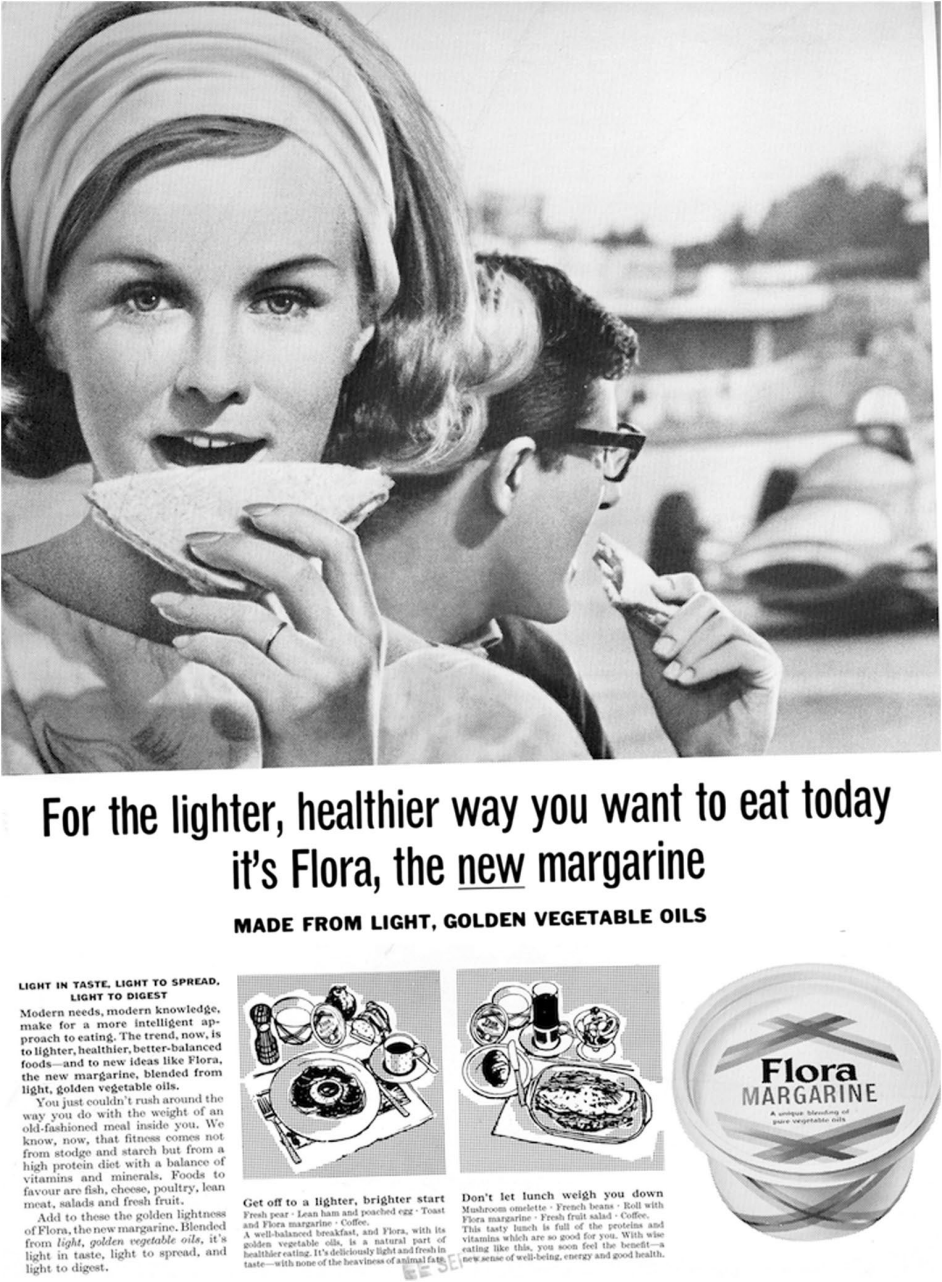
‘For the lighter, healthier way you want to eat today—it’s Flora, the new margarine’, advertisement, September 1964. Source: Reproduced with kind permission of Unilever [from an original in Unilever Archives].

Unilever first introduced Flora to the British margarine market in two test sites—Bolton and Brighton from 1964 to 1966. This test phase was accompanied by press advertising portraying the benefits of the product framed in terms of natural goodness. The message of Flora’s test site advertisements in 1964 exclaimed that ‘modern needs, modern knowledge, make for a more intelligent approach to eating. The trend now, is to lighter, healthier, better-balanced foods—and to new ideas like Flora’ (Figure 1).^28^ The focus in this copy-line on modernity, lightness and health revealed that from the outset Unilever identified health as a key element in establishing its unique selling point. By doing so Unilever advertising closely linked the tenets of healthy lifestyles, risk avoidance and personal behaviour change to a specific type of food consumption. By encouraging potential purchasers to consume a product with identifiable health benefits—vegetable-based, polyunsaturated fats—Unilever altered the relationship between edible fats and the diet–heart disease link. Historians of public health and food, academic nutritionists and food policy experts have all identified this appropriation of health by the food industry as an important factor in the food economy during the twentieth century.^29^ In particular, they have noted the accelerated rise of functional foods or ‘nutraceuticals’ in the marketplace since the 1980s.^30^ Such functional foods aimed to maintain health and create conditions beneficial to reducing the risk of ‘diseases of affluence’. But the development of food products that delivered health benefits and informed consumers on how food consumption could help prevent disease was formulated long before the manufacture of food products with specific health-inducing additives such as plant sterols or omega-3. While, the linking of food and health was nothing fundamentally new in the post-war period, what remained innovative about Flora advertising was the focus on chronicity (not nutritional deficiency), disease prevention and the identification of polyunsaturates as the *single* health-inducing ingredient.^31^ In this respect, Flora advertisements presented consumers with ‘scientific’ details about the possible links between diet and heart disease, representing one way in which epidemiologically focused understandings of risk and individual responsibility segued from the laboratory and into the household.

Unilever’s linkage of Flora with a specific health claim was incorporated into its visual advertising material from late 1969 and by June 1971 the company declared that ‘there is now so much evidence to support the theory that dietary fats are important in heart diseases, that the general public should know more about it’.^32^ This statement revealed the company’s explicit objective—to position themselves as public health educators. To this end, Unilever developed a policy for launching Flora to ‘publicise the connection between dietary fats and atherosclerosis as widely as possible’.^33^ Internal memoranda reveal that a central principle was ‘conscience as food manufacturers … to induce the public to buy what [they] genuinely believe to be best for them’.^34^ By moving into the realm of health education in this way, Unilever, through the Flora brand, responded to the emergent scientific research that linked high fat diets (including those containing butter) with disease. From the outset, therefore, the development of Flora was not only as a value-added butter substitute. Rather, Unilever marketed Flora as a health-inducing product with the aim of ‘launch[ing] a high polyunsaturated fats margarine as a service to the public’.^35^

## Flora claims health

Unilever’s initial marketing campaign for Flora was tentative in advertising the product in terms of CHD or blood cholesterol lowering. Instead, these initial advertisements referenced wide-ranging connotations of healthiness as a general goal of modern life. It was not until full nationwide distribution began in 1968 that product advertising appropriated the marketing potential of the health claim with greater commitment and exactitude.^36^ As outlined above, the initial advertising for Flora because of its test site focus was directed at Bolton and Brighton, and these advertisements revealed important points of continuity with previous margarine campaigns introduced by Unilever. Their later approach to advertising centred on visualising the male body to sell female-centric modes of shopping had not yet taken hold. Rather, there remained a visual focus on the traditional wife and mother as the object of the advertising ‘look’ although these test site advertisements provided another framework for analysing the relationship between Flora advertising, notions of modernity and the ways in which men and women were imagined to sell health.

Building on the lifestyle-orientated marketing closely associated with Unilever’s previous high-end margarine products, particularly Blue Band during the 1950s, Flora’s initial regional advertising in newspapers and magazines followed a similar visual pattern. The major departure from the aesthetic of the Blue Band advertisements of the 1950s was the repositioning of eating away from home and into the outside environment. Flora was represented visually as the picnic margarine spread, the reliable alternative to butter for modern, middle-class pursuits. As shown in Figure 1, the woman was the central element of the photographed scene. In the middle ground, her partner, also eating, was looking away from the viewer, his eyeline acting as a leading device to direct the viewer’s eye to the background image of a racing car ‘speeding’ around a track. The man’s stance, turned away from us, visually suggested that he is not ‘engaged’ with the message of the campaign—the healthy choice. Instead, the woman, staring directly into the camera, linked her consumption of Flora-spread sandwiches with the ‘healthier way you want to eat today’. Therefore, this visual arrangement suggested that the woman was the visual and textual target (the ‘you’ of the copy line) for this advertisement. Yet, rather than emphasising the contemporary role of women as wives and mothers, as with traditional margarine advertisements, the text used the rhetoric of modern lifestyles, the idea that ‘modern needs, modern knowledge, make for a more intelligent approach to eating’ in order to persuade viewers/readers to purchase Flora.

Certainly, the copy-line of this newspaper advertisement revealed the centrality of dietary advice in targeting prospective purchasers of Flora. As a ‘healthier, better-balanced food’ with ‘the lightness you want’, Flora was visually and textually portrayed as ‘modern’ and healthy. By doing so, Unilever attempted to disrupt the commonly held belief that butter had important protective properties.^37^ In this case, polyunsaturated fats were isolated as *the* new and modern component of Flora margarine with Unilever committed to a ‘high poly-unsaturated fats margarine as a service to the public … to create the image of Unilever as a progressive, forward-looking authority on questions of fat and diet’.^38^ While such products have often been criticised for appropriating a reductionist approach to disease prevention through diet in conjunction with issues of taste, cost and the selling of particular lifestyles, Unilever’s Flora was exceptional as a butter substitute in adopting a health education function on a national level from the outset.^39^

By adopting the tenets of epidemiological public health, marketers explained disease in terms of risk factors (and how certain foods combated this), and Flora advertisements contributed to the widespread dissemination of individualised tools for prevention. Roberta Sassatelli has traced a similar development within post-war fitness culture, emphasising the complexity of the gym as not just a site for achieving the perfect body, but also as a locale where a vast array of meanings and identities are negotiated and re-negotiated.^40^ She suggested that the local environment is of particular importance in shaping both wider cultural values and the ideals of the fit, slim body, itself coded as a symbol of individual choice and social worth. Margarine consumption in Britain had been greatly affected by the impact of butter rationing during the Second World War. Associated with austerity, margarine had faced an uphill struggle in attempting to regain market share against butter during the period of decontrol. Thus, margarine manufacturers and advertisers needed to re-imagine their product not in terms of scarcity but as a symbol of a new, post-war, healthy society. As a result, Flora quickly became bound up with normative injunctions, inviting ‘modern’ health-conscious individuals to take responsibility for their bodies—to feed them foods beneficial to health and thereby enable them to invest in body presentation for their self-constitution.^41^

**Figure 2.**
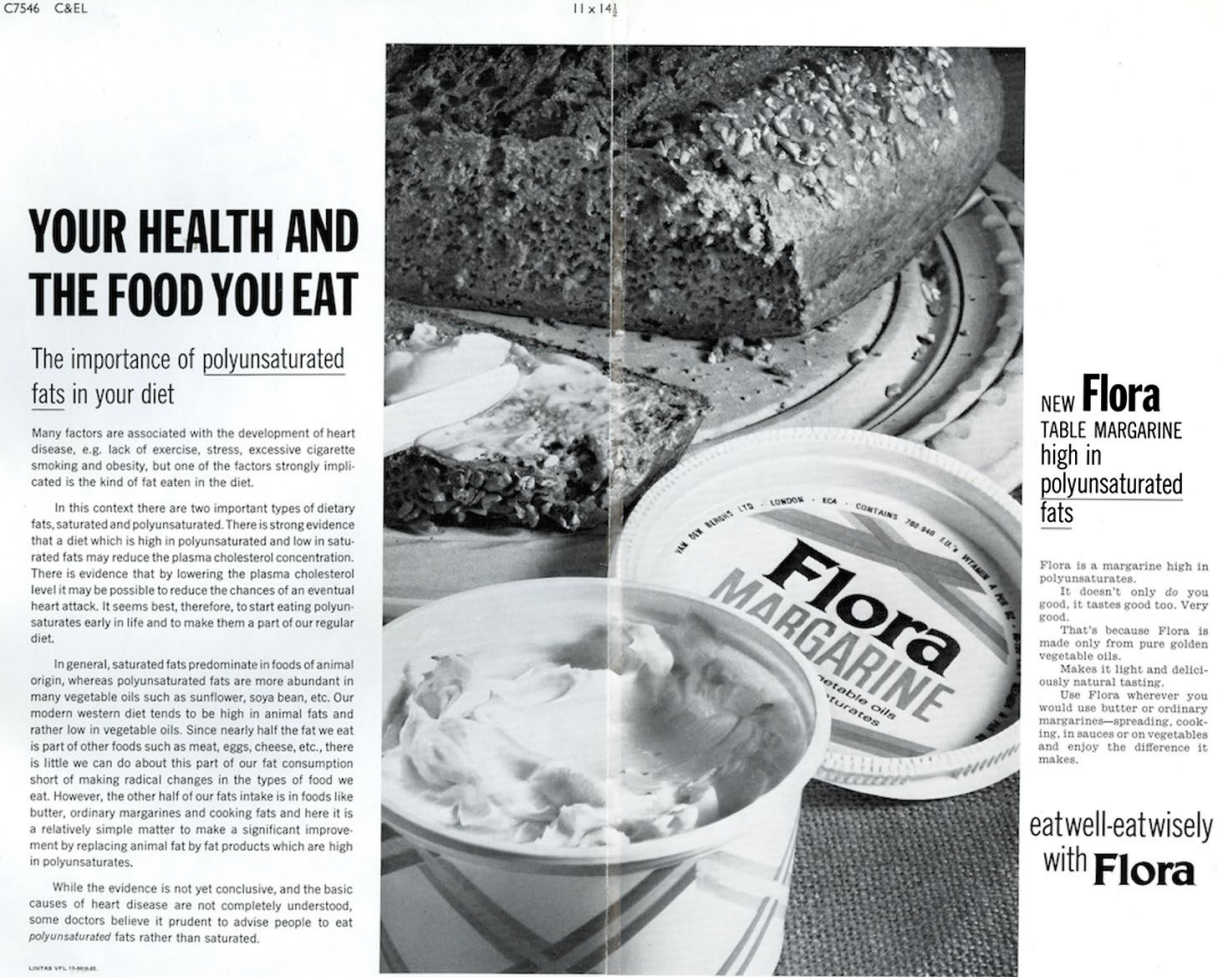
‘Your Health and the Food You Eat’ advertisement, 1968. Source: Reproduced with kind permission of Unilever [from an original in Unilever Archives].

Because of this changing social and consumer base, the visual depiction of women was re-negotiated as part of Flora’s advertising campaigns. In these initial Flora advertisements, women were still identified as the central agents for encouraging behavioural change within the realm of health and nutrition. But this straightforward interpretation of the role of women in relation to food consumption in the home was complicated by the high rates of male mortality from heart disease and other chronic conditions. Men were suffering from higher rates of ‘diseases of affluence’ and consequently Unilever’s visual tools of selling Flora had to adapt to a changed reality. Moving from their more traditional tried and tested visual composition of family units and female figures as the main targets of advertising, Unilever altered their advertising strategy for Flora following its nationwide expansion in 1968. The principal change was that it now began to explicitly emphasise the health benefits of the product for heart health rather than references to general healthiness or ‘lightness’ (Figure 2).

To do so, they briefly removed human figures from their visual promotional material altogether. Instead, they focused on the margarine itself, with close-up images of the Flora tub and an accompaniment such as brown bread. The brand now appropriated a health education style of address with a large section of the advertisement dedicated to textually outlining the links between diets high in saturated fats and the risk of heart disease. Unilever claimed that because Flora contained high levels of polyunsaturated fats, which it argued could help lower cholesterol and therefore reduce the risk of a heart attack, it was beneficial to health. This advertisement, while seemingly visually conventional (and conforming to contemporary publications that conveyed health risk in terms of behaviour) was original and inventive because it functioned primarily as an advertisement—to encourage viewers to buy a product—and not purely as a tool of health education—to persuade viewers to alter their personal behaviours. By bridging a gap between advertising as a social ill and health education as a positive tool for the public good, Unilever, through Flora, was assuming a voice of authority with regard to heart disease risk.^42^ Pertinently, the visibility of Flora as a brand within the visual and textual components of this advertisement was less emphasised than the detailed text about ‘Your Health and the Food You Eat’. It was only on the right-hand side of the advertisement that Flora was advertised in terms closely associated with marketing. Consequently, the visual component of the advertisement was largely supportive—clearly showing one possible inclusion of Flora margarine in the daily diet. The placement of newly gentrified fibre-rich brown bread, as opposed to the ever-popular mass produced white bread, aligned Flora with a shift in food consumption towards healthier options. Indeed, the presence of the new tag line ‘Eat well-eat wisely with Flora’ reinforced this notion that Flora was at the forefront of a new healthy-eating trend in post-war Britain.

The advertisement assumed the look and scientific journalism style of a broadsheet newspaper. Its use of headlines, sub-headings and blocked text divided by one central linked image was already customary within the world of print journalism and considering the highly medicalised information that the advertisement was trying to convey, Unilever opted for a notable departure from Flora’s household-structured advertising forerunners. Thus, as this Flora advertisement was placed in newspapers during early 1968, it integrated into the wider, informative broadcast context of the daily newspaper. The advertisement aimed to explain the effects of large quantities of animal fat in the body in relation to heart disease specifically. It used particular, detailed scientific vernacular such as ‘plasma cholesterol concentration’, while embedding this specificity within general health advice regarding fat intake and the potential health benefits of polyunsaturates. Notably, the textual portion of the advertisement closed with ‘while the evidence is not yet conclusive, and the basic causes of heart disease are not completely understood, some doctors believe it prudent to advise people to eat *polyunsaturated* fats rather than saturated’. By admitting that the scientific evidence remained tentative during the 1960s, Unilever adopted a ‘truth-telling’ stance. It strongly suggested that by increasing the amount of polyunsaturated fats in individual diets it might be possible to avoid heart health issues in the future, ‘It seems best … to start eating polyunsaturates early in life and make them part of our regular diet’. Therefore, from the launch of Flora nationwide in 1968, Unilever committed itself to the dissemination of health information pertaining to diet and health to sell their product.

In establishing Flora as a health brand, Unilever was carefully re-negotiating the institutional and cultural formation of food risk and responsibility. Adopting a language of self-care, it designed this polyunsaturated margarine as one source of health authority within post-war British public health consumerism. While Unilever’s central aim was to sell the Flora product, their employment of the rhetoric of health education widens the historical understanding of health education in post-war Britain. While selling a ‘healthy’ product has a different overall aim than governmental attempts at selling ‘healthy behaviours’, the inclusion of conceptions of risk, personal responsibility and choice demonstrates how Unilever was functioning, at least in terms of language, in selling a product within the model of contemporary health education. Unilever’s health claim for Flora became increasingly important to its distinctiveness as a margarine product, while its advertising campaigns became an educational force in their own right.

Therefore, the historical process by which popular understandings of disease prevention was publicised can be explored in relation to the health marketing of food products during the late twentieth century. Not only were notions of ‘selling’ health central to programmes of popular education at the behest of government but they also formed important components of marketing agendas by the food industry.^43^ In doing so, Unilever and their Flora brand helped to link the production of scientific knowledge about nutrition and their social facets with the promotion and individualised application of such knowledge within the environment of the home. It was because Unilever created a special place for Flora within the relationship between emergent governmentally instituted modes of health (and eating) behaviours, and the creation of consumer-focused health foods that it occupied an important position in the development of health food marketing as a ‘responsible action’ in post-war Britain.^44^ No longer was the process of health education pertaining to food one-way and government-led through public health campaigning, whether at the local or national level. It was now dynamic and fluid, with information, ideas and images traded among different audiences including, but not limited to, scientists, practising physicians, the food industry, the science press and government institutions whilst accommodating competing and disparate views, becoming as it did part of the working hypothesis of everyday life.^45^

## ‘Stop: Ought he to be eating Flora?’

**Figure 3.**
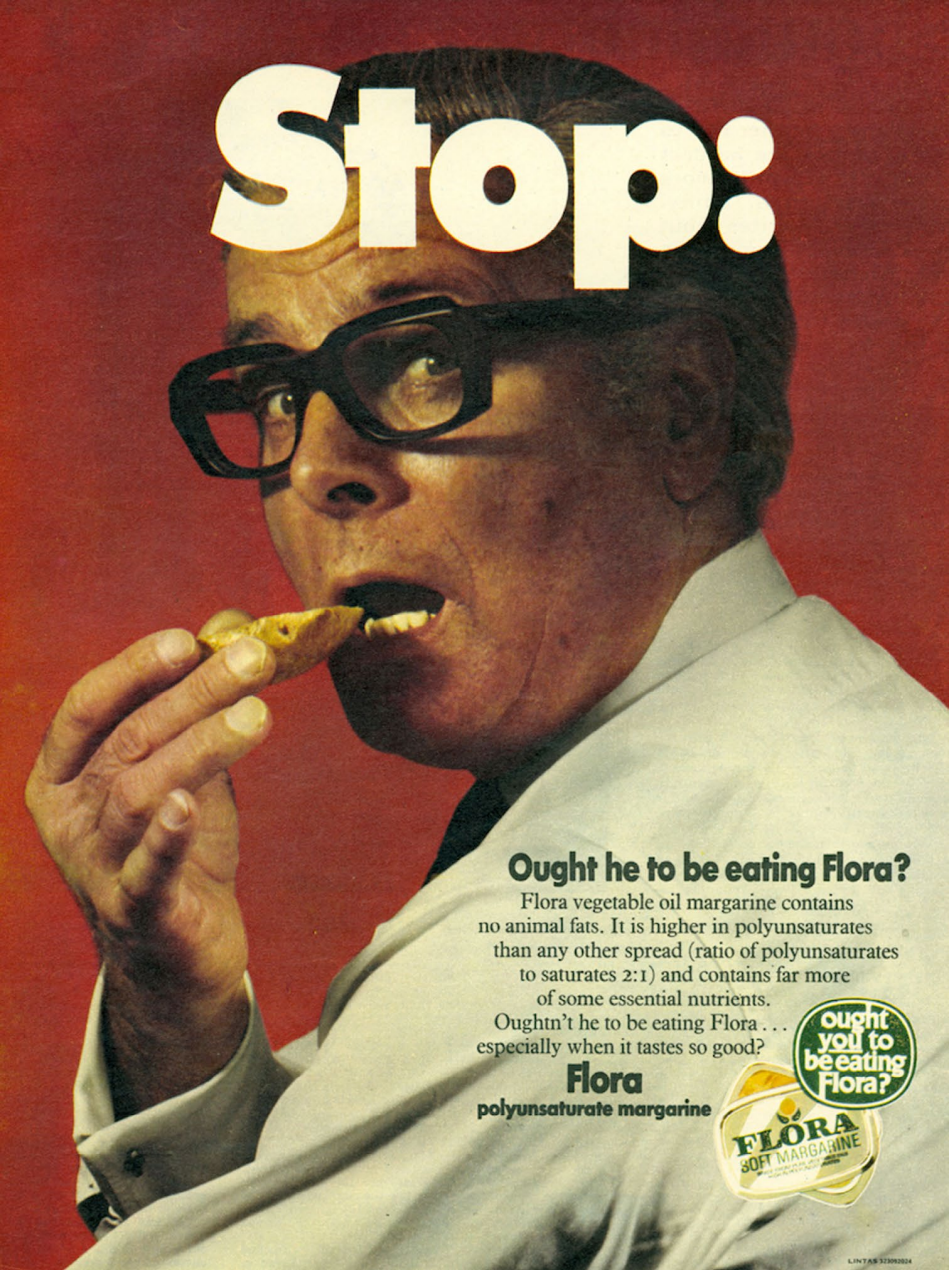
‘Stop. Ought he to be eating Flora?’ June 1971—version that appeared in Readers Digest. Source: Reproduced with kind permission of Unilever [from an original in Unilever Archives].

With the dual decision to launch a new advertising campaign for Flora centred on the at-risk male figure, coupled with the decision to launch a Flora Information Service during 1970 and 1971, Unilever renegotiated accepted modes of gendered advertising and the social role food brands could play beyond the confines of commercial life. In the first campaign to do so, the poster advertisement for ‘Stop: Ought he to be eating Flora’ (Figure 3) combined these selling and educative functions. The visual component of the poster displayed a foregrounded middle-aged man about to eat a piece of bread. His torso was turned away from the viewer but his face was twisted back towards us, as if suddenly caught in an act that is, if not unacceptable, then at least discouraged. By portraying the male figure turning his face towards the viewer, a level of hesitation was established. The word ‘Stop’, which covered the top of the man’s face, was emblazoned in large white font, prompting a reconsideration on his part. A man in motion, in the process of lifting a morsel of bread to his mouth was frozen in mid-movement. He was represented considering or being reminded of the benefits of healthy eating by adding Flora to his daily diet. The bright red background emphasised the male figure, rendering his locational setting unimportant to the tenets of healthy eating. The photographic style in which the man was presented recalled the everyman, the realistic and relatable potential sufferer of chronic disease. The advertisement was produced in colour, rather than black and white, reflecting both its reproduction within a magazine (as opposed to a newspaper), which had already embraced colour images within its advertising pages, and the wider shift on the part of large corporations to advertise in colour in order to ‘draw on different type[s] of “psychological theme”[s]’^46^ As Stefan Schwarzkopf argued, colour advertisements, particularly in consumer magazines, ‘worked on the incitement of fears, jealousies or largely hidden sexual desires’.^47^ Therefore, by displaying a generic, middle-aged man—at the highest risk from CHD in 1970s Britain—this advertisement aimed to incite fears, or at least caution, to change a very particular eating habit. Not only was it implicitly suggested that margarine was healthier than butter—‘Flora vegetable oil margarine contains no animal fats. It is higher in polyunsaturated fats than any other spread’—it was proposed that Flora was healthier than other standard margarine brands because of its high content of health-inducing polyunsaturated fats. Similarly, an apparent division was outlined between animal and vegetable fats, with the health benefits of the latter being central to the marketing strategy of Flora. This approach conformed to a good deal of medical research in the early 1970s that was emphasising the role of saturated animal fats in CHD and the potential benefits of polyunsaturated fatty acids in counteracting this.^48^

The visual and textual formulation of the advertisement established the brand itself as the central expert and imparter of health information. Thus, the food manufacturer was seen to speak for itself, based on the research of food technology and food science experts and not through the medium of a general health professional, for example.^49^ The male figure in the Flora advertisement was voiceless. He was shown as a motionless, opinion-less object of the ‘modern’ health project, compliant with its health mission—the individual involved (or soon to be involved) in healthy personal behaviours. The real health expert that this advertisement sought to establish was Flora, the brand that is ‘Everything you need to make Flora the kind of advice that people are likely to take’.^50^

By suggesting ‘Ought he to be eating Flora?’ the textual support, typed over and onto the main image, created a dialogue between Flora as a health educator, and the male figure as the important agent of change. Notably, this visual straightforwardness was complicated by the use of the third person in the textual element of the advertisement. This was an unusual grammatical choice considering that it was aiming to change both the shopping and eating habits of ‘you’ the viewer. The use of ‘he’ in this case automatically gendered Flora as a margarine for men specifically and yet insinuated that the ‘you’, the viewer of this advertisement might not have been male but female—the wife who wishes that ‘he’, her husband, would consume more beneficial dietary fats. While the visual figure of the man already insinuated this, the textual support confirmed it. This was a decisive move by Unilever. The advertising campaign altered the visual tradition of women as central figures in margarine and butter advertising and instead only obliquely referenced their widespread societal role as homemakers responsible for food choice. In their place, this Flora campaign made men not just the central visual component, but the only visual component—the lone man as the representative man at risk from developing CHD.

By coding the individual at-risk from CHD as personally and totally responsible (even if the obligation for purchasing still lay with women) for their own health outcomes, Unilever’s Flora advertisements were visually re-constructing bodies in ways not dissimilar to government-sponsored health education initiatives during the same period. Contemporary governmental health education posters, largely produced by the Health Education Council, were likewise utilising notions of persuasion to ‘sell’ new health behaviours. Focused on single-issue advice campaigns, the Council repeatedly utilised images of the body to clearly link disease prevention with personal bodily behaviours.^51^ Building upon an established visual language and vernacular in discussing health, Unilever marketed Flora as *the* brand through which these principles of a modern public health system could be achieved. Unilever’s chief objective in launching Flora as an unconventional ‘expert’ within a wide range of health actors that were commenting on health, diet and disease during this time was to attach the brand closely to the gradual scientific realisation of the connection between polyunsaturated fats and heart disease. Because polyunsaturates were an important new element in the composition of Flora margarine, it was logical to strengthen the marketing of the polyunsaturated fats element in line with this shift at a time when the scientific link between saturated fat and heart disease was gaining increased media attention.^52^ During the ‘Stop’ campaign, the polyunsaturated claim remained moderate, ‘confined to the statement that margarine contains poly-unsaturated fats and, in very general terms are believed to have beneficial effects on one’s health’ combined with other authenticated health claims and advertisable qualities such as flavour or texture.^53^ As a later ‘Stop’ advertisement made clear: ‘Some people may buy Flora for its flavour, the easy spreading or simply because it’s fashionable. It doesn’t matter, just so long as they stay with it. Because Flora is made to do you good’.^54^

During the post-war period, the broader societal value placed on affluence, self-sufficiency and personal choice further directed consumers to adopt a prophylactic approach to their health through diet and fitness.^55^ The combination of government campaigns and food industry marketing initiatives that emphasised the role of preventive health, individual responsibility, lifestyle choice and behavioural change within the context of food, diet and fitness expressed a wide-ranging understanding of health in relation to individualised and personal body management practices.^56^ In this context, the ability to ‘add value’ to products was paramount for food manufacturers. As Flora so aptly demonstrated, by creating a health-consciousness around the brand, Unilever was able to capitalise on the rising consumer interest in healthy behaviours (although only easy, supplementary behaviours rather than restrictive practices) in part formed by the contemporaneous public health mandate and the focus in consumer culture on attaining certain beauty ideals. Its emphasis on the individual at-risk as their identified market base allowed the company to utilise the Flora Information Service to further establish the brand as both a health product and a voice of nutritional and scientific authority. This duality allowed Unilever to adopt an innovative approach to its Flora advertising, which integrated with wider trends in food marketing concerned with selling ‘natural’ products.^57^

While slimming and fitness cultures were largely directed at women during the post-war period, from the 1980s these have been increasingly targeted at men through magazines, self-help books and the televisual and cinematic media. Such developments created a general narrowing of gender differences in terms of shaping the ideal, healthy body through lifestyles and social expectations. It contributed to the normalisation of images of the ‘built’ male body in popular culture as a positive personal attribute associated with strength, perseverance and self-control. However, while these endeavours to create the idealised male more generally have largely failed, in terms of the rising rates of obesity in contemporary Britain, they have not prevented the tenets of beauty and the pursuit of the lifestyle-orientated healthy body from pervading consumer culture and the mass media. Thus, in terms of the development of a health advertising culture in post-war Britain, it was the perception of the healthy male rather than the sobering reality that persisted as an important marker of modernity.

## ‘The Margarine for Men’

Following on from the perceived success of the ‘Stop’ advertising campaign, Unilever’s marketing strategy for Flora further aligned the product with visualisations of the male body. So for its next advertising campaign the at-risk middle-aged man was replaced by the young, healthy, fit male body—the body engaged from an early stage in upholding the tenets of prevention. In this way, Unilever reconstituted Flora as not just a foodstuff high in healthier fats but as a product that had particular preventative functions. As Unilever made clear, Flora advertisements ‘portrayed the goodness and benefits of the product [and] its role in the maintenance of heart health for all, but particularly men’.^58^ To this end, Unilever launched ‘The Margarine for Men’ campaign in 1976. While the visual components of these advertisements suggested that male purchasers were themselves important targets for Unilever, internal memoranda revealed that a more complicated visual construction process was at work. Interestingly, it was Unilever’s intention that this campaign ‘target … primarily women and show … a man’s torso’.^59^ It utilised complex (if outwardly simplified) visual imagery to render masculinity and the male body as attractive and beautiful not just to men but to women too.

**Figure 4.**
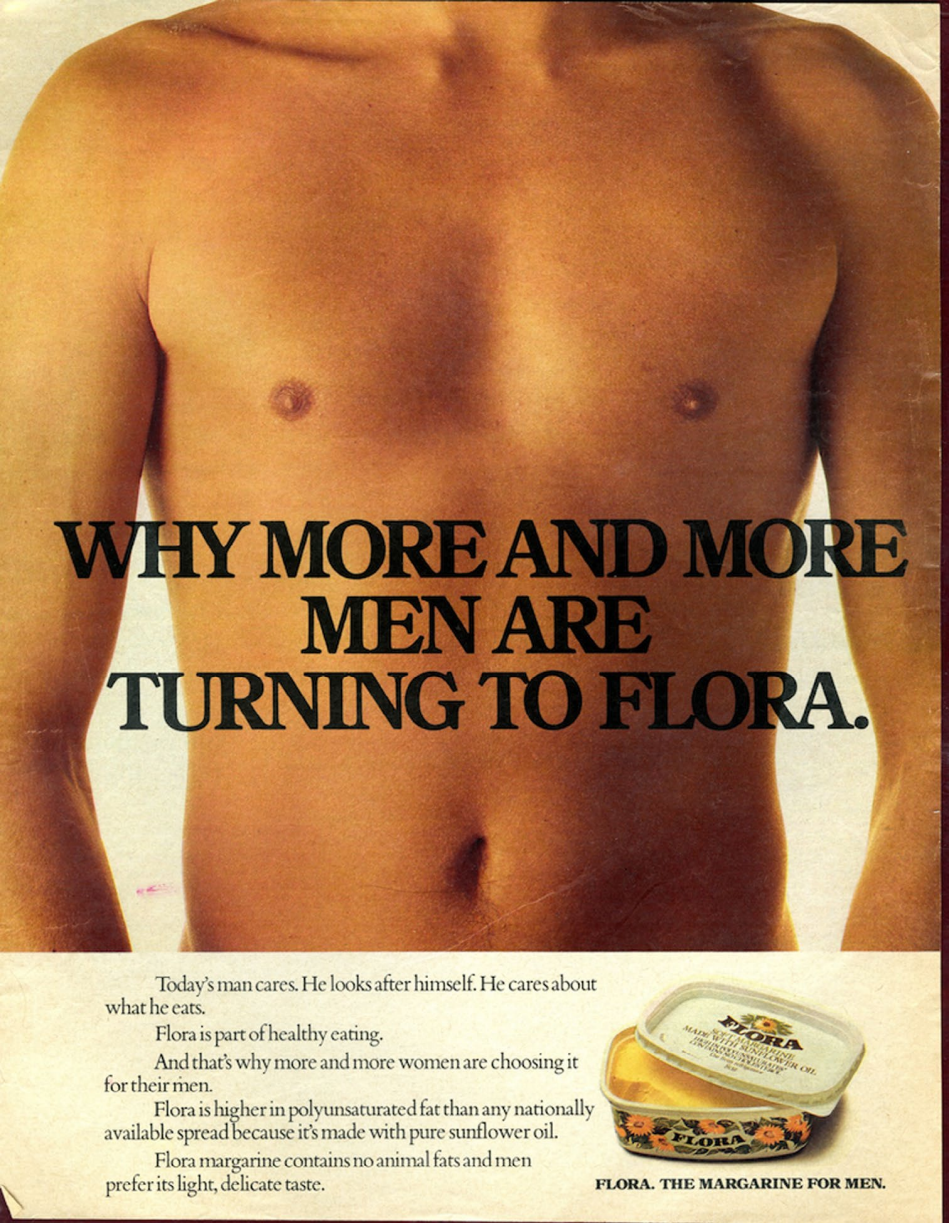
‘Why More and More Men are Turning to Flora’, advertisement, 1978. Source: Reproduced with kind permission of Unilever [from an original in Unilever Archives].

As shown in Figure 4, this advertisement from 1978 displayed a close-up static photograph of a man’s toned, tanned torso from the navel to the neck. The visual emphasis on the male chest dovetailed with the emergence of the male body as an object of mainstream consumption in fashion, cinema and advertisements.^60^ Susan Bordo has examined this re-emergence of the nude male body in the mid-to-late twentieth-century United States, linking it to the development of a ‘pure consumerism’ that recognised the marketing (and purchasing) potential of male fitness and beauty.^61^ Advertising similarly advanced culturally contingent notions of bodily beauty and the specific ways this was visible or inscribed *on* the body and therefore discernable by the viewer. In this advertisement the flat, toned chest and stomach of the male figure in conjunction with the tanned, hair-free torso publicised notions of what healthiness ‘looked’ like and implicitly suggested that apart from eating Flora, both regular exercise and fitness were key means of achieving this ideal. Interestingly, the head of the man was cut off, existing beyond the frame of the advertisement. By portraying this figure of apparent objective male attractiveness (and by extension, healthiness) as faceless, personality-less, the torso itself was able to become both desirable and personal for the ‘everyman’, allowing the viewer to impose individualised meanings and desires upon the image and thus ‘fill in the blanks’.

As the campaign developed during the 1970s, Unilever associated the margarine with the creation and maintenance of happy and content personal relationships. Firstly, this facilitated a movement into the realm of the family, while retaining a visual commitment to depictions of the healthy male body. They continued to represent the family man and the woman shopper as the epitome of ‘modern’ life. The committed, healthy, fit father and husband was visually coded as the essence of Flora’s health-promoting mission. For example, another advertisement showed a young boy standing next to his father, who was largely out of the frame, presumably shaving. The young boy applied shaving foam to his chin in emulation of his father with the tag line reading ‘He’s just like his Dad’. It established the ‘Flora man’ as an important role model for younger male children.^62^ It suggested that by emulating his father, and including healthy Flora margarine in his diet, this young boy could assume the habits and behaviours of his father (and likewise become a ‘Flora man’). Yet, the advertisement suggested that the opposite was also true. The phrasing suggested either that the father was responsible for bequeathing the tenets of healthy eating habits to his son or that the son should follow Dad’s example by eating healthy foods chosen, bought and cooked by mum. After all the advertisement ended with the question ‘How soon will all your men be Flora men?’ In this respect, the implicit educator in healthy eating behaviours was still the traditional responsible mother. The representation of Flora as one part in a litany of behavioural and social elements that passed from parent to child established Flora as desirable within a very different context to the pervious Flora advertisement (Figure 4).

Between 1981 and 1983, Unilever moved away from male only visual images in their advertising material with the implied women shopper (who by giving their husbands Flora, transformed them into ‘Flora men’) re-emerging within the images of the brand. By 1986 ‘The margarine for men’ tag line in these advertisements was altered to ‘The margarine for you’ recognising not only the place of women within the Flora brand success, but also that women themselves were increasingly being identified as at-risk from CHD.^63^ Rather, Unilever returned to older, more traditional forms of margarine advertising centred on ‘captur[ing] a genuine maternal/caring spirit with which mothers could identify’ and in doing so submitted Flora as a margarine brand for the whole family rather than the ‘at risk’ man alone.^64^ It was also during the 1980s that the Flora product range grew considerably to include cooking oils, salad dressings and a cheese spread as well as a diversified margarine range that included Flora Extra Light and Flora Reduced Salt and which received a more equal gender balance in advertising terms.^65^ Throughout the 1990s, advertising for their original product continued to centre on health and the importance of polyunsaturates. It was not until 1996 that the company applied a functional food message to the brand by including the message: ‘As part of a healthy diet can help to lower cholesterol and to maintain a healthy heart’, on all Flora packaging.^66^ This marked the first real use of the health claim label on a margarine product in Britain that directly linked consumption with heart health in pragmatic terms. While Unilever had previously applied scientific language to promote Flora products, the application of a prophylactic role to the product stemmed from a wider consumerist commitment to developing functional foods that could be advertised and sold, based on very specific claims to the health benefits of consumption. It was within this wider context that Unilever developed and launched Flora ProActiv, their first cholesterol lowering spread containing plant sterols.

## Conclusions: ProActiv and the promotional health claim

Throughout the post-war period, commercial marketing activities have played an important and often overlooked role in disseminating health information. This article has begun the process of incorporating commercial advertising into the historical narrative of post-war consumerism and public health as important artefacts that attempted to educate the British public about health risks. With the launch of Flora ProActiv in the year 2000, margarine had been completely transformed from a product perceived inferior to butter to a product with health benefits at complete odds with butter. The Flora Information Service did much to facilitate the inclusion of short health education pieces centred on diet and heart disease as marketing tools, therefore ensuring that scientific knowledge—whether undeniable or not—was disseminated to the public in new ways, distinct from governmental efforts. This approach proved durable. Unilever continually reinforced their health education role in many of Flora’s subsequent advertisements during the 1990s and indeed with the launch of Flora ProActiv, the provision of an information service was again a central component of its advertising campaign.

I have primarily focused on diet, using Unilever’s Flora brand as an innovative and new mode of health education based on health claims, added value and educative marketing. In doing so, I examined a number of Flora advertisements and demonstrated how Unilever represented gender norms, the body and healthy lifestyles as the essence of what it meant to be ‘modern’ during the post-war period. As medical science increasingly identified saturated fats as having an adverse effect on the likelihood of developing CHD, Unilever seized on social responses by engaging at-risk individuals as key agents of behavioural change. In doing so they enlisted the ideology of the ‘new public health’ to commodify the beautiful, fit and healthy body.^67^ By commercialising the techniques for disciplining the body through food (conforming to epidemiologically determined health behaviours), Unilever utilised the same rhetoric of individual responsibility for health and disease prevention that the government promoted. Thus, they emphasised the personal strategic dimension of health claims in selling food in the post-war period.^68^

By providing an alternative view of health education dissemination, which dovetailed with, but was not centred on contemporaneous governmental initiatives, I suggest that there was a distinct continuity between state-led efforts and the commercial approaches of multinational corporations such as Unilever. In this way, the development of health education in Britain pertaining to food and heart health was more complex and layered than is often understood. Buying health became a commodity in the second half of the twentieth century as concepts of lifestyle choice, behavioural modification and individual risk were subsumed into the commercial world of the food industry. Visual depictions of nutrition and diet supported and extended such notions of individualism in health. Consequently not only did food become medicalised but medicine also became increasingly receptive to the persuasive force of food advertising and promotion. Nutritional information became disconnected from the medical domain of the doctor/patient relationship and instead was firmly placed within the sphere of the consumer, albeit the educated one. The later launch of Flora ProActiv, containing plant sterols illustrates the continuing importance of the health claim to selling food, while the capacity of consumers to purchase and consume a range of marketable products centred on lowering cholesterol complicates the divide between eating and treating.

## Funding

This work was supported by the Wellcome Trust [grant number 096712/Z/11/Z].

## Disclosure statement

No potential conflict of interest was reported by the author.

## Notes on contributor

***Jane Hand*** is a research fellow for the Wellcome Trust Senior Investigator Award 'The Cultural History of the NHS' in the Centre for the History of Medicine at the University of Warwick. She researches public health and health education in Britain with a focus on health campaigning, chronicity and the place of prevention.

## Notes

1. Levene, “The Meanings of Margarine,” 145–65; and Burnett, *Plenty and Want*, 304–5.

2. The Seven Countries Study and the Framingham Study were particularly influential in emphasising the role saturated fats might be playing in the incidence of CHD.

3. ‘Healthy’ or ‘healthier’ margarine spreads from all margarine manufacturers became staples of margarine market by the close of the century. Polyunsaturates was just one-way manufacturers created unique selling points. Other brands would capitalise on the ‘Mediterranean diet’, marketing spreads enriched with olive oil (for e.g. Olivio—now Bertolli), while others still would advertise their vegetable oil content as the main healthy additive (Clover).

4. For more on Unilever see: Jones, *Renewing Unilever: Transformation and Tradition*.

5. Levene, “The Meanings of Margarine,” 145–65; Van Stuyvenberg, *Margarine*; Ball and Tilly, “The Menace of Margarine,” 488–98.

6. Levene, “The Meanings of Margarine,” 146.

7. For example see: Berridge and Loughlin, “Smoking and the New Health Education in Britain”; Cooter and Stein, “Coming into Focus”; and Jordanova, *Sexual Visions*.

8. Slicer, “Unilever and Economic Power.”

9. Ibid.

10. Swett, *Selling Under the Swastika*; and Ciarlo, *Advertising Empire.*

11. Berridge, “Public Health in the Twentieth Century, 1945–2000,” 195–210.

12. Berridge, *Marketing Health*, 132–16; and Bufton, “British Expert Advice on Diet and Heart Disease,” 25–148.

13. Blue Band, Summer County and Echo were perhaps the most successful in being advertised in terms of advertising, texture and taste.

14. Interestingly, it was the Dutch medical community that first approached Unilever about the possibility of the development of a margarine with health attributes distinct from butter and other margarine brands then on the market. Unilever Archives “The Evolution of Margarine,” UNI/1974/1.

15. An initial product test phase was carried out in Bolton and Brighton in 1964, with a nationwide launch following in 1968. Unilever Archives, *Flora Brand History*, Un-catalogued.

16. Flora differed from Blue Band compositionally. It contained polyunsaturated fats made from vegetable oils rather than, for example, whale oil.

17. Unilever Archives, Untitled, VBF/13/1/2/2/2/2.

18. Horrocks, “The Business of Vitamins,” 234–58; Zweiniger-Bargielowska, “The Culture of the Abdomen,” 239–73; Offer, “Body Weight and Self-control in the United States and Britain,” 79–106; La Berge, “How the Ideology of Low Fat Conquered America,” 139–77; Stearns, *Fat History*; and Levenstein, *Paradox of Plenty*.

19. Hesse-Biber, *Am I Thin Enough Yet?*; Wolf, *The Beauty Myth*; Seid, *Never Too Thin*; Bordo, *Unbearable Weight*; and Featherstone, “The Body in Consumer Culture,” 18–33.

20. Sturken and Cartwright, *Practices of Looking*, 82.

21. Rose, “Temperate Heroes,” 177–86; Arnold and Brady, *What is Masculinity?*; Francis, “The Domestication of the Male?” 637–52; Segal, *Slow Motion*; and Scott, “Gender,” 1053–75.

22. Kirkham and Thumim, *You Tarzan*, 12–14.

23. For more on governmental health campaigns see: Hand, “Visualising Food as a Modern Medicine.”

24. Unilever Archives, *Formulating Unilever Policy*, A4 5350.

25. Unilever Archives, *Fats, Heart Disease and Unilever*, A4 5350.

26. Anon, “The Story of the Flora Project,” Early publications included the recipe booklet Eating for a Healthy Heart, a cholesterol and saturated fats counter and booklets on protecting the whole family from CHD.

27. Carroll, “Corporate Social Responsibility,” 268–95.

28. Quote from Figure 3: ‘For the Lighter, healthier way you want to eat today—it’s Flora, the new margarine’, advertisement (Unilever Archives, MD/AL 107/1-B10309), September 1964.

29. Nestle, *Food Politics*; Lang and Heasman, *Food Wars*; Heasman and Mellentin, *The Functional Foods Revolution*; and Scrinis, “Functional Foods or Functionally Marketed Foods?” 541–5.

30. See: Fitzsimmons, “Oh What Those Oats Can Do,” 56–99; and Scrinis, “Nutritionism and Functional Foods,” 269–91.

31. Apple, *Vitamania*; and Kamminga, “Axes to Grind,” 83–100.

32. Anon, *Van den Berghs and Jurgen News*, No. 10, June 1971, p. 1.

33. Anon, “Fats, Heart Disease and Unilever”, Unilever Archives, Port Sunlight, A4 5350.

34. Ibid.

35. Marketing Flora as a public service links to a wider interpretation of corporate social responsibility. See: Jones, *Renewing Unilever*.

36. Anon, *Marketing Division Guardbook*, Unilever Archives, Port Sunlight, UNI/GF/MD/AL/1/176.

37. Levenstein, *Paradox of Plenty*, 195–212.

38. Unilever Archives, *Fats, Heart Disease and Unilever: Putting the Policy into Practice 1964*, A4 5350.

39. Fitzsimmons, “Oh What Those Oats Can Do,” 56; Heasman and Mellentin, *The Functional Foods Revolution*, 3–33; and Scrinis, “Nutritionism and Functional Foods,” 269–91.

40. Sassatelli, “Interaction Order and Beyond,” 227–8.

41. Synnott, *The Body Social*; and O’Neill, *Five Bodies*.

42. Schwarzkopf, “They Do It With Mirrors,” 135–6; Moscucci, “The British Fight Against Cancer,” 356–73; and Toon, “Cancer as the General Population Knows It,” 116–38.

43. Yakult was the first global health food brand that claimed a value-added health benefit. It was launched in Japan in 1935 and expanded internationally in the postwar period.

44. Unilever Archives, *Flora Brand History*, Un-catalogued.

45. This approach is greatly indebted to the work of Nancy Tomes in *The Gospel of Germs*.

46. Schwarzkopf, “They Do It With Mirrors,” 139.

47. Ibid.

48. A search of the British Medical Journal for example reveals in excess of one thousand entries that discuss saturated fat and CHD between 1970 and 1974. Furthermore, the DHSS Report, Diet and Coronary Heart Disease, published in 1974, emphasised excess dietary fat as one risk factor for CHD with the potentially beneficial role of polyunsaturated fatty acids is cautiously discussed (although no recommendation for increasing the amount in the diet is made in the Report).

49. The use of the GP as the voice of authority in commercial advertising was perhaps most successfully employed for advertising the ‘More Doctors Smoke Camels’ campaign of the late 1940s and early 1950s.

50. Unilever Archives, *Heart*-*Risk: People and Polyunsaturates, 1970*, Flora/Go Organic Box.

51. The notion of persuasion was key to health education in from the late 1960s, especially as post-war advertising theory, imported from the United States, facilitated the design and distribution of campaigns that used the tenets of contemporary advertising to promote governmental health advice. Notably, the Health Education Council contracted the advertising agency Saatchi & Saatchi, who were largely responsible for creating anti-aesthetic health education movement in postwar Britain.

52. Unilever Archives, *Fats, Heart Disease and Unilever: Conclusion 1964*, A4 5350.

53. Unilever Archives, *Fats, Heart Disease and Unilever: Putting the Policy into Practice 1964*, A4 5350.

54. Unilever Archives, *Stop. Ought You to be Eating Flora? advertisement c.1970*, Flora/Go Organic Box.

55. The so-called ‘diseases of affluence’ suggests the fluidity with which disease, affluence and lifestyle were constructed as interlinked.

56. Zweiniger-Bargielowska, *Managing the Body*, 176–7, 31–4; and Berridge, *Marketing Health*, 132.

57. Levenstein, *Paradox of Plenty*, 195–7.

58. Unilever Archives, *Fats, Heart Disease and Unilever*, A4 5350.

59. Unilever Archives, *Flora Brand History*, Un-catalogued.

60. See: Bordo, *The Male Body*, 168.

61. Ibid.

62. Interestingly, there is no evidence of daughters or young girls being identified as important actors in assuming healthy habits that they would grow up possessing. Although it wouldn’t be until later in the decade that women would be visually reinstated in the visual advertising of Flora at all, and when they did so, they tended to focus on the adult woman.

63. As Unilever made clear: ‘This change acknowledged the fact that it was not just men who were at risk from heart disease—women were just as vulnerable—and recognised the fact that the success of “The Margarine for Men” campaign had widened brand usage’. See: Unilever Archives, *Flora Brand History*, Un-catalogued.

64. Unilever Archives, *Flora Brand History*, Un-catalogued.

65. Ibid.

66. Ibid.

67. For more on the ‘new public health’ see: Berridge, *Marketing Health*.

68. Sassatelli, “The Commercialization of Discipline,” 396–411; and Sassatelli, “Interaction Order and Beyond,” 227–48. For more on the rise of the patient-consumer see: Mold, *Making the Patient*-*Consumer*.
